# From targeted *SCN1A* analysis to whole exome sequencing: clinical utility and novel genetic findings in a Hungarian paediatric epilepsy cohort

**DOI:** 10.1007/s00439-026-02867-w

**Published:** 2026-08-29

**Authors:** Renata Szalai, Agnes Till, Krisztina Galimurka, Zsolt Banfai, Anna Zsigmond, Kinga Hadzsiev

**Affiliations:** Medical School, Department of Medical Genetics, University of Pecs, Pecs, Hungary

## Abstract

Epilepsy represents a highly prevalent neurological disorder with a significant genetic component, particularly implicating ion channel genes, including *SCN1A*. In this study, 431 individuals with heterogeneous paediatric-onset epilepsy phenotypes were assessed at the Department of Medical Genetics, University of Pécs between 2018 and 2024. Genetic investigations employed Sanger sequencing, targeted epilepsy gene panels, whole exome sequencing, and multiplex ligation-dependent probe amplification for *SCN1A* copy number analysis. Thirty-six pathogenic or likely pathogenic *SCN1A* variants were identified, including 15 variants which have not been reported previously. Furthermore, 9 novel variants were detected in 12 additional epilepsy-associated genes. Diagnostic yield was proportional to the breadth of genomic interrogation. WES analysis revealed 6 novel variants in 19 genes. These findings underscore the considerable genetic heterogeneity of epilepsy and demonstrate the clinical utility of gene panels and WES, particularly in complex phenotypes. The identification of novel variants enhances molecular understanding and facilitates more precise genotype-phenotype correlations, reinforcing the value of comprehensive genomic diagnostics in epilepsy management.

**Supplementary Information:**

The online version contains supplementary material available at https://doi.org/10.1007/s00439-026-02867-w.

## Introduction

Epilepsy is a neurological disorder of the central nervous system characterized by a predisposition to generate epileptic seizures and their neurobiological, cognitive, psychological, and social consequences (Fisher et al. 2005; Scheffer et al. 2017). Seizures arise from abnormal, excessive, and synchronized neuronal activity in the cerebral cortex, leading to transient disruption of normal brain function and a wide spectrum of clinical manifestations (Fisher et al. 2005; Blumenfeld 2010). Epilepsy affects approximately 65 million people worldwide, with higher prevalence in low- to middle-income countries (Fiest et al. 2017). Its aetiology is highly heterogeneous, encompassing structural, genetic, infectious, metabolic, and immune causes, which may overlap (Scheffer et al. 2017; Rastin et al. 2023). Genetic factors play a particularly important role in early-onset epilepsies, including epileptic encephalopathies, where both *de novo* variants and, less frequently, chromosomal abnormalities or metabolic disorders have been identified (Morrison-Levy et al. 2021; McTague et al. 2016). In some cases, structural abnormalities such as cortical malformations are also present, often with an underlying genetic background (Leventer et al. 2008; Guerrini et al. 2014; Sah et al. 2021).

Severe epilepsies involve diverse mechanisms, including channelopathies, synaptic dysfunction, metabolic defects, and transcriptional dysregulation (Rastin et al. 2023; Oliver et al. 2014; Noebels 2015). Advances in molecular genetics, particularly next-generation sequencing (NGS), have significantly improved diagnostic yield by enabling parallel analysis of multiple genes (Mefford 2015). As the costs of whole exome sequencing (WES) and whole genome sequencing (WGS) continue to decline, these comprehensive approaches are rapidly becoming the primary diagnostic tools worldwide, allowing both targeted analysis of known genes and the discovery of novel disease-associated variants (Sabau et al. 2025; Mefford 2015; Demos et al. 2019).

Numerous genes have been associated with epileptic phenotypes. Among them, *SCN1A* is one of the most frequently implicated, particularly in Dravet syndrome and Genetic Epilepsy with Febrile Seizures Plus (GEFS+) (Zuberi et al. 2011). Other important genes encode proteins involved in ion channel function, synaptic transmission, and neuronal regulation, disruption of which can alter the balance between excitation and inhibition (Guerrini et al. 2014).

Epilepsy-related genes can be broadly categorized into epilepsy genes, neurodevelopment-associated genes, and epilepsy-related genes based on their predominant clinical presentation (Wang et al. 2017; Zhang et al. 2024). While these categories often overlap and exist on a continuous phenotypic spectrum in certain syndromes, such as Dravet syndrome or *KCNQ2*- and *PCDH19*-related encephalopathies, seizures typically represent the primary clinical feature. In other conditions (e.g. Rett syndrome, tuberous sclerosis complex), epilepsy occurs as a secondary manifestation within a broader neurodevelopmental context. Developmental and epileptic encephalopathies (DEEs) represent the most severe end of this spectrum, characterized by early-onset seizures and significant, often progressive comorbidities (Guerrini et al. 2023).

In this retrospective study, we evaluated the diagnostic findings of a single-centre paediatric epilepsy referral cohort examined between 2018 and 2024. Our primary aim was to assess the clinical contribution of an evolving genetic testing workflow, from targeted *SCN1A* sequencing to epilepsy gene panels and whole exome sequencing. Because testing was phenotype-driven and the cohort was enriched for *SCN1A*-associated presentations, we interpreted the findings as real-world diagnostic outcomes rather than population-representative estimates of paediatric epilepsy genetics in Hungary.

## Materials and methods

### Patient and sample recruitment

Between 2018 and 2024, a total of 512 patients with paediatric-onset epilepsy phenotypes were referred to the Department of Medical Genetics at the University of Pécs. The epilepsy patients were referred for genetic testing by paediatric and adult neurologists based on suspected genetic aetiology. Patients were enrolled based on a paediatric onset (under 18 years), the availability of comprehensive clinical records, and a diagnosis of treatment-resistant epilepsy according to international guidelines.

**Fig. 1:**
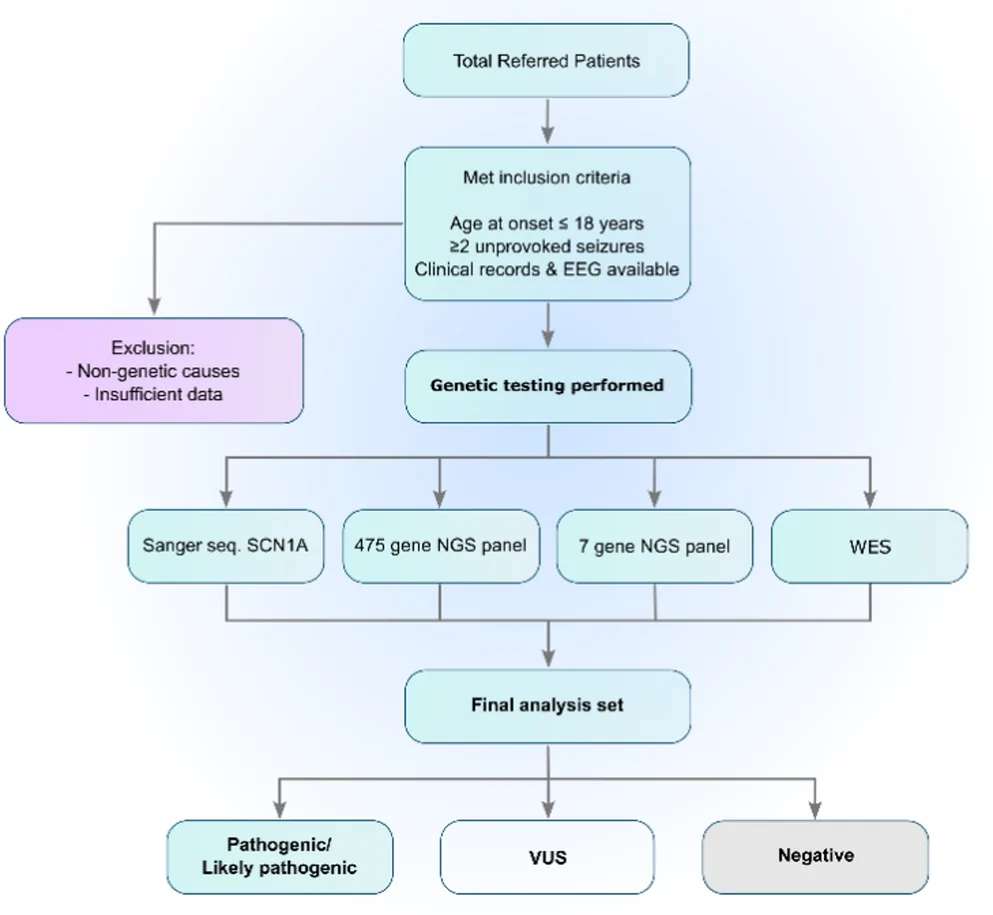
Flow chart of patient selection process, genetic testing, and final outcomes in the studied epilepsy cohort

**Fig. 2:**
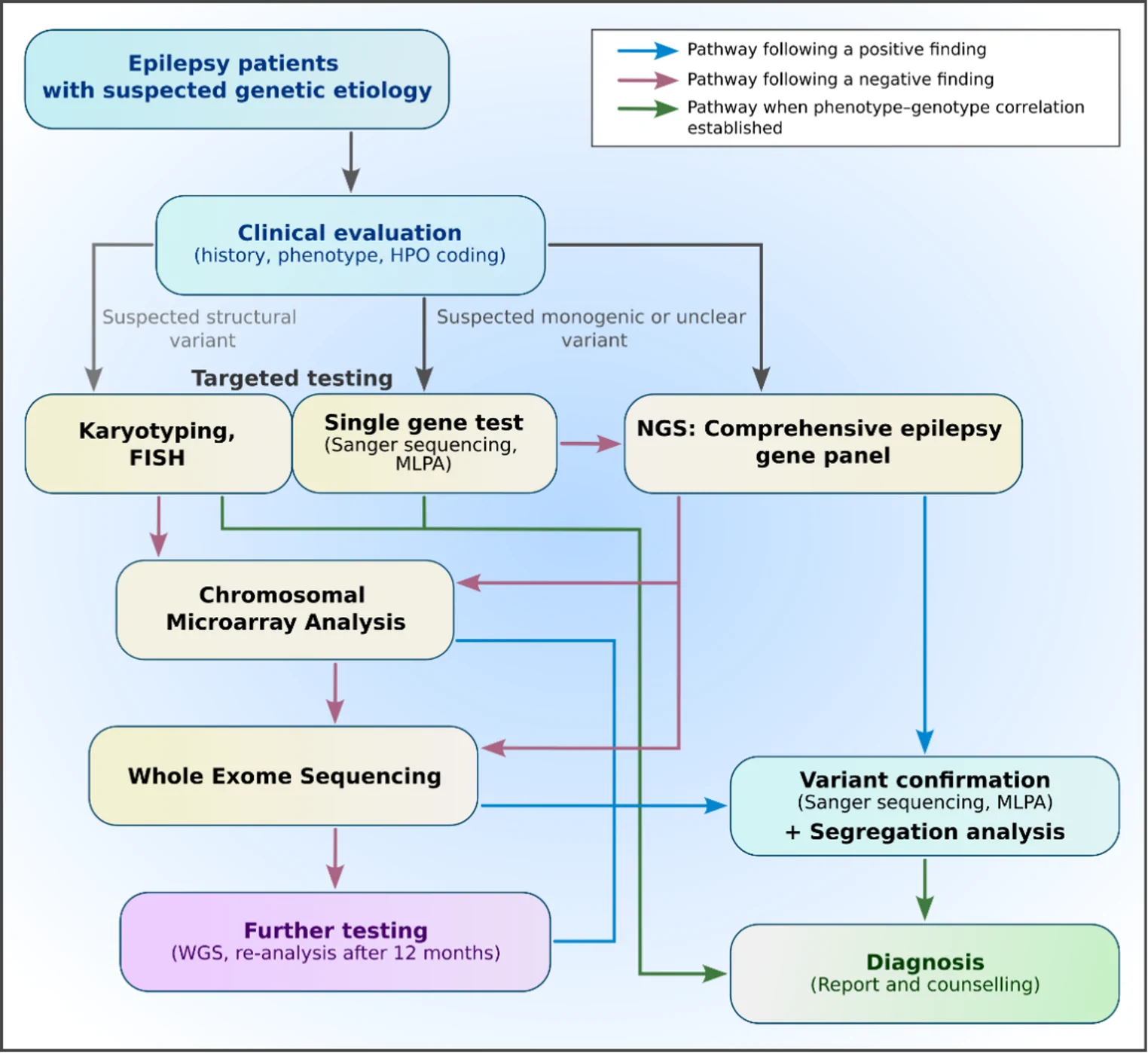
Diagnostic algorithm for epilepsy patients with suspected genetic aetiology

Out of the 512 patients, 431 met the inclusion criteria. Patients were excluded from the study based on explicit exclusion criteria, including non-genetic aetiology or insufficient clinical data. Specifically, patients with a history of perinatal brain injury (such as intracranial hemorrhage or hypoxia) and those with post-infectious conditions (e.g., following meningitis or encephalitis) were excluded. Thus, 431 epilepsy patients participated in molecular genetic testing in the final cohort. DNA samples from these patients were examined using Sanger sequencing and NGS-based techniques (epilepsy gene panel, comprehensive epilepsy gene panel, and exome sequencing) (Fig. 1). Segregation analysis was performed for all families where parental DNA was accessible. Full-gene sequencing of *SCN1A* was performed in cases characterized by infantile onset and seizures triggered by fever or infection, specifically targeting phenotypes consistent with Dravet syndrome and GEFS+. Clinical characteristics of these participants, including age, sex, age of onset, and age at diagnosis are presented in Table 1. Patients were referred from different regions of Hungary; however, the cohort should not be considered population-representative. It represents a clinically selected, referral-based diagnostic cohort enriched for patients with suspected genetic epilepsy and, particularly in the earlier phase of the study, for SCN1A-associated phenotypes The clinical geneticist determined the genetic testing workflow after a detailed review of the medical history and a thorough physical examination. The diagnostic algorithm applied for the examination of epilepsy patients in our institute presented in Fig. 2. The study was approved by the Ethics Committee of the University of Pécs (Protocol 8770-PTE 2021.).

After genetic counseling, written informed consent was obtained from participating members prior to their inclusion in the study. During the collection and analysis of DNA samples and processing of the accompanying clinical and personal data the guidelines and regulations of the Helsinki Declaration in 1975 and the currently operative national regulations (Hungarian law; XXI/2008) were followed. Genomic DNA was extracted from peripheral blood, using E.Z.N.A. Blood DNA Maxi extraction kit (OMEGA^®^, Bio-tek, Inc., GA, USA). The concentration and purity of extracted DNAs were measured with the NanoDrop 2000 spectrophotometer (Thermo Fisher Scientific, Waltham, MA).

### Generation of sequence data

Direct sequencing was performed to examine the coding exons of *SCN1A* gene and to segregation analysis, furthermore, to confirm the presence of variants identified by NGS panel and exome sequencing. Sanger sequencing was performed on an Applied Biosystems 3500 Genetic Analyzer (Applied Biosystems, Foster city, CA, USA) (capillary electrophoresis, BigDye Terminator chemistry) according to the manufacturer’s instructions (https://documents.thermofisher.com/TFS-Assets/LSG/manuals/4401661.pdf).

Since 2020, next generation sequencing-based targeted custom epilepsy gene panel analysis was performed by QIAGEN QIAseq library kit (QIAGEN GmbH, Hilden, Germany) and Illumina MiSeq (Illumina, San Diego, CA, USA) sequencing technology. From 2022 a comprehensive epilepsy panel (475 genes) was used for the identification of pathogenic variants in patients with epilepsy by IDT xGen DNA Library Prep EZ Kit (Integrated DNA Technologies, Inc. Coralville, IA, USA) and Illumina NovaSeq 6000 (Illumina, San Diego, CA, USA) sequencer device. Exome libraries were prepared using the Twist Human Core Exome Kit (Twist Bioscience, San Francisco, CA, USA) and IDT xGen DNA Library Prep EZ Kit (Integrated DNA Technologies, Inc. Coralville, IA, USA). Sequencing was performed on Illumina instrument according to the manufacturer’s protocol, using paired-end 150 bp reads.

WES was performed on an Illumina NovaSeq 6000 (Illumina, San Diego, CA, USA) platform using standard sequencing-by-synthesis (SBS) chemistry. This technology relies on cyclic reversible termination, where fluorescently labeled nucleotides are incorporated by DNA polymerase and detected by imaging at each cycle, followed by cleavage of the terminator to allow the next base addition. The NovaSeq system employs patterned flow cells to enable massively parallel sequencing with high throughput, generating paired-end 2 × 150 bp reads with an average yield of 8–12 Gb per sample, and providing average coverage above 120×.

### Analysis of sequence data

Raw sequencing reads obtained from WES and targeted gene panels were processed using an in-house standard NGS analysis workflow. Reads were aligned to the human reference genome (GRCh37) with BWA-MEM, followed by sorting and indexing with SAMtools. Duplicate reads were marked using GATK4 MarkDuplicates, and Base Quality Score Recalibration (BQSR) was performed with GATK4 BaseRecalibrator. Variant calling was conducted with GATK4 HaplotypeCaller in accordance with GATK Best Practices. Variant annotation was performed using ANNOVAR, SnpEff, and SnpSift, which provided comprehensive information for each variant, including genomic position, gene context, variant consequence, zygosity, depth of coverage, and quality metrics, as well as population frequency data (gnomAD, 1000 Genomes, ExAC), predicted functional effects, and clinical significance from databases such as ClinVar and OMIM. The resulting variant lists were systematically reviewed in spreadsheet format, and putative pathogenic variants or other variants of interest were further evaluated using online tools such as Franklin (Genoox) and VarSome, which provide ACMG guideline–based classifications and additional population and functional evidence. Variant interpretation was performed according to ACMG/AMP guidelines, using automated implementation in Franklin (Genoox) and VarSome, which integrate ACMG rules with population, functional, and clinical databases (Richards et al. 2015). Regarding *SCN1A* variants, the classification was performed according to ACMG/AMP guidelines with ClinGen Epilepsy Sodium Channel Variant Curation Expert Panel (*SCN1A*-specific) v2.0 specifications. Softwares applied in NGS data processing and interpretation are presented in Appendix 1. Moreover, in silico prediction tools, including SIFT, PolyPhen-2, MutationTaster, PROVEAN, DANN, CADD, REVEL, MetaSVM, MetaLR, MutationAssessor, FATHMM, GERP++, PhastCons and PhyloP were used.

### Statistical analysis

Descriptive statistics were applied, including absolute numbers and percentages. Diagnostic yields were calculated as the proportion of positive cases among tested individuals. No inferential statistical tests were performed. Statistical reporting follows the SAMPL (Statistical Analyses and Methods in the Published Literature) guidelines.

### Multiplex ligation-dependent probe amplification (MLPA)

MLPA assays were performed for screening large deletions or duplications in *SCN1A* gene using the commercially available SALSA MLPA kits P-137 (MRC-Holland, Amsterdam, The Netherlands), which contained probes for all exons of *SCN1A*. According to the manufacturer’s instructions, a total of 100–200 ng of genomic DNA of each patient and the same amount of three control genomic DNA was used for hybridization. Amplification products from each MLPA assay were separated by capillary electrophoresis on an ABI 3130 Genetic Analyzer (Life Technologies, USA) and the results were analysed using Coffalyser software (MRC‐Holland, Amsterdam, The Netherlands). Each MLPA signal was normalized and compared to the corresponding peak area obtained from the three control samples. Deletions and duplications of the targeted regions were suspected when the signal ratio exceeded 30% deviation.

## Results

Molecular genetic testing of epilepsy patients was introduced at our department in 2012. Initially, examinations were limited to the Sanger sequencing of *SCN1A* gene. Former results from 2012 to 2017 were reported by Till et al. (2020). Till and colleagues examined 183 patients with epilepsy; we extended their studies over the next seven-year period. In subsequent years, the investigation was expanded to a custom epilepsy NGS panel (7 genes) and then to a comprehensive epilepsy gene panel (475 genes) analysis depending on the phenotype of patients (Appendix 2). To investigate more complex cases - syndromic patients with epilepsy as the leading symptom - WES method was used to identify the variant underlying the disease. Between 2018 and 2024, further 431 patients with a spectrum of paediatric-onset epilepsy were examined. Sanger, gene panel and exome sequencing identified a total of 30 novel variants. These were not reported previously in the literature and databases.

**Fig. 3:**
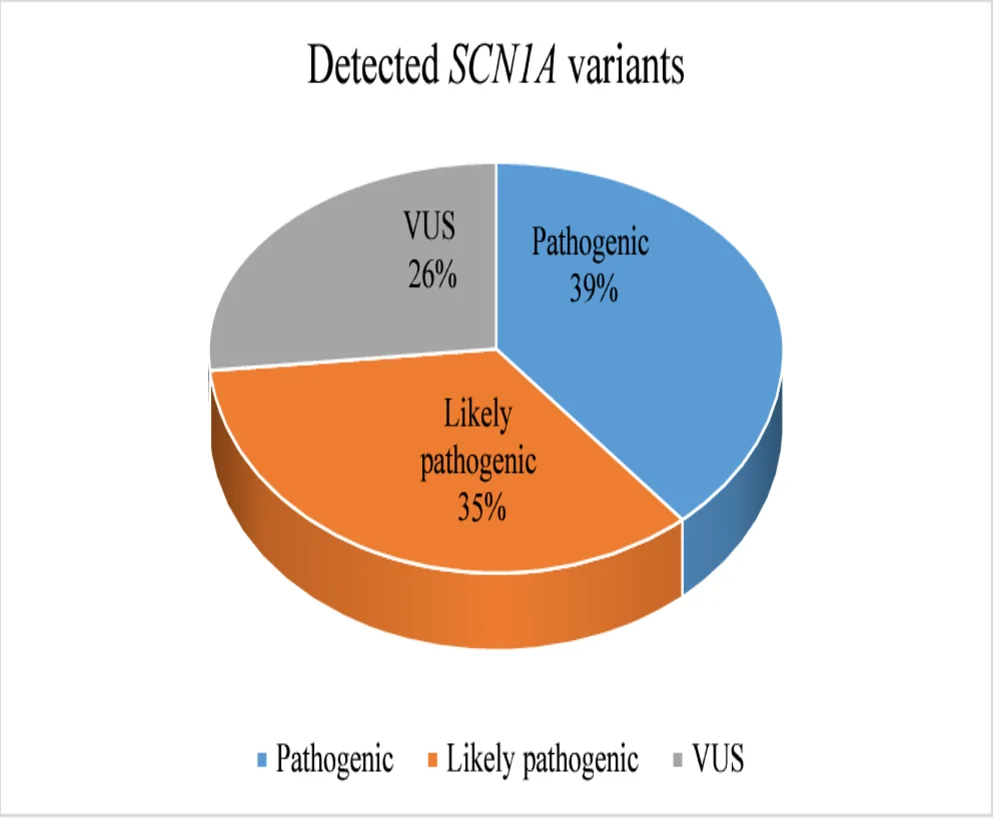
Distribution of identified *SCN1A* variants with classification in our epilepsy patient cohort

**Fig. 4:**
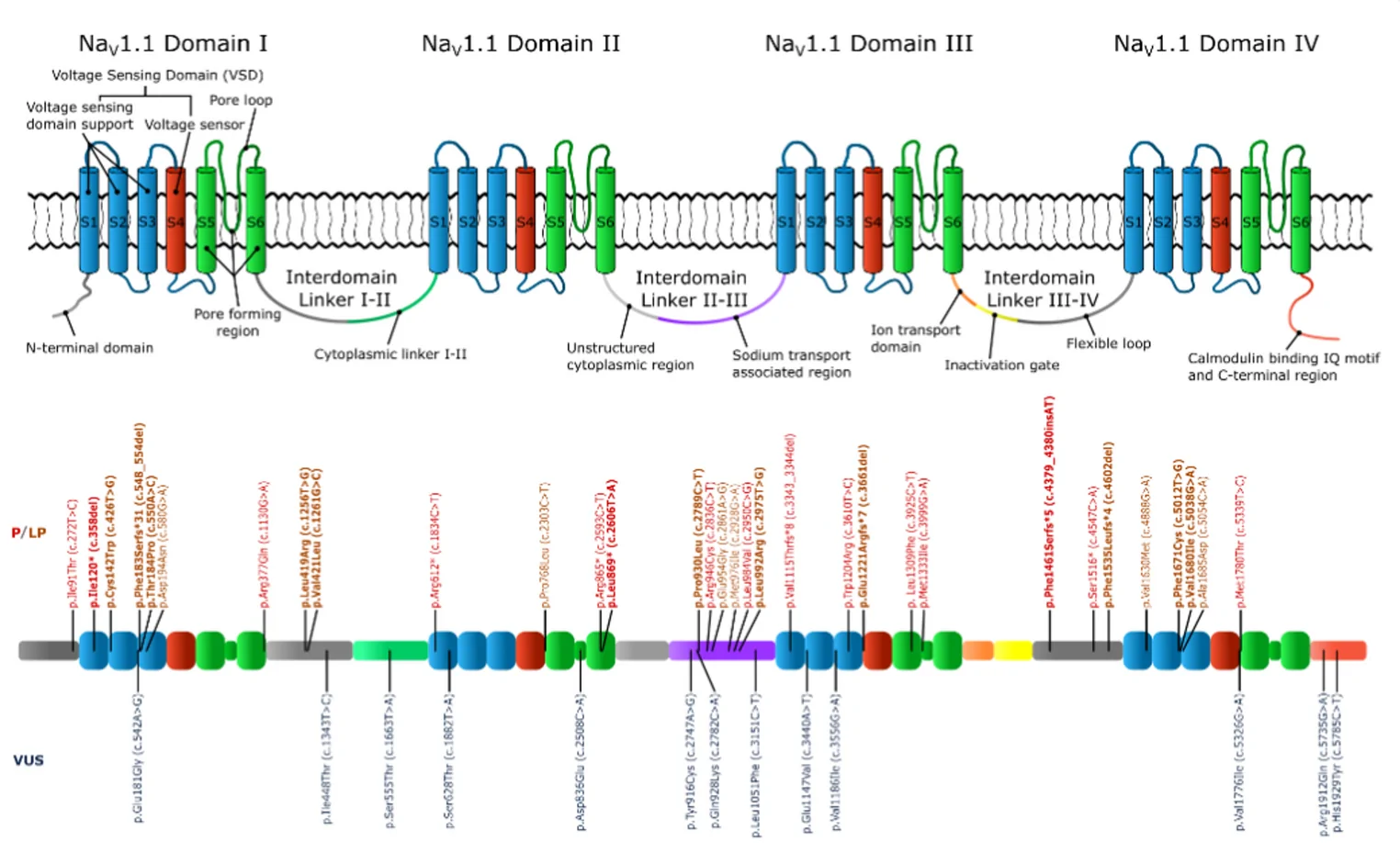
Schematic representation of the *SCN1A* protein structure and mapped germline variants

**Fig. 5:**
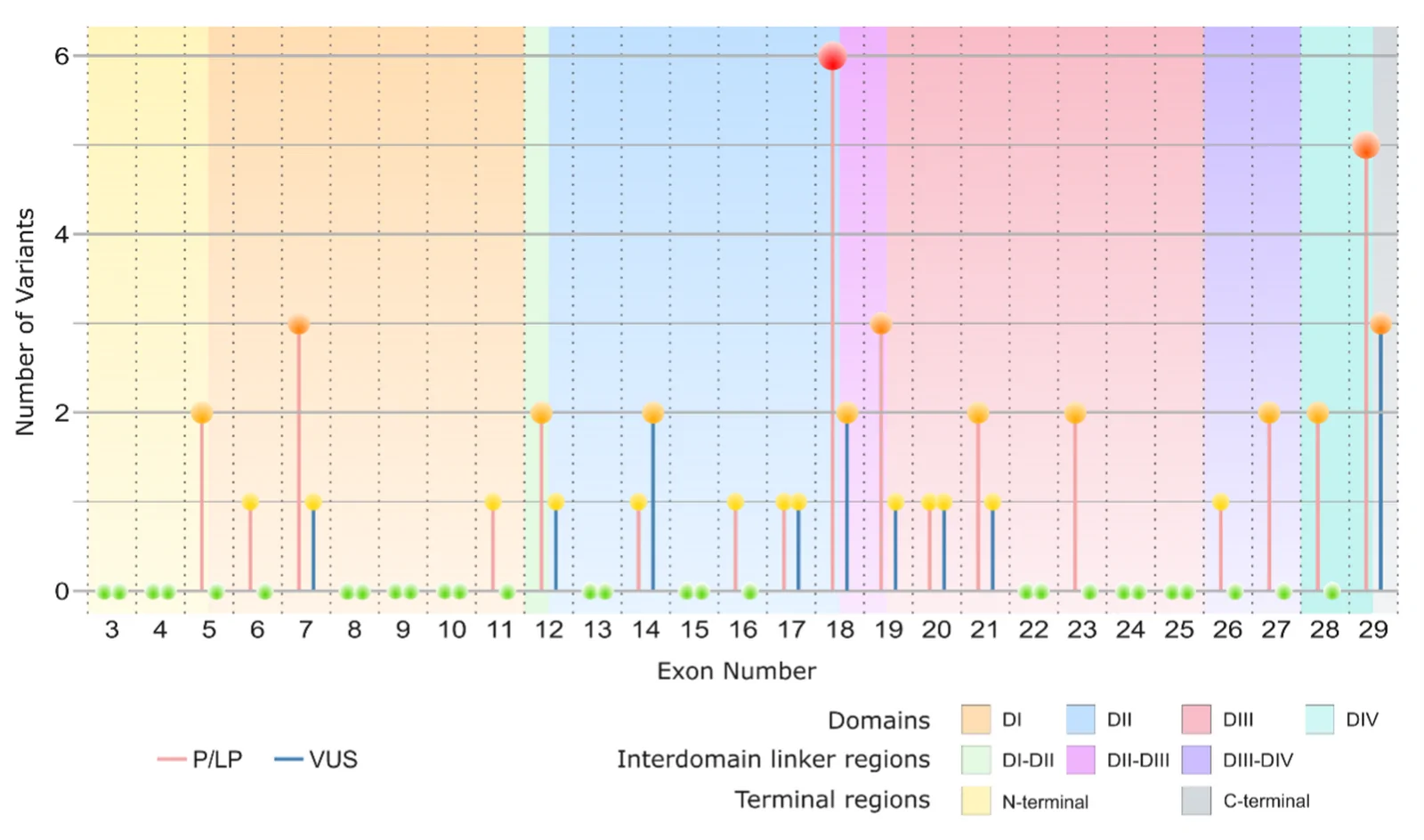
Exon-based visualization of *SCN1A* variant counts and classifications

Of the 431 epilepsy patients, 76 subjects were positive - pathogenic or likely pathogenic - for Sanger and NGS-based panel sequencing tests. Detected pathogenic and likely pathogenic variants in *SCN1A* (NM_001165963.4) gene are summarized in Table 1. The distribution of identified *SCN1A* variants is presented in Fig. 3. A total of 49 *SCN1A* variants were identified (Figs. 4 and 5), out of these 19 were pathogenic and 17 were likely pathogenic, 13 were found to be VUS. Among 17 likely pathogenic variants, 11 were novel, have not been reported in the literature previously. The distribution of novel likely pathogenic *SCN1A* variants was as follows; 8 missense and 3 frameshift variants. Identified *SCN1A* variants with uncertain significance (VUS) are summarized in Table 2. During the second stage of *SCN1A* screening, sequence-negative patients with Dravet syndrome and GEFS+ phenotype were tested with MLPA method. All MLPA tests were negative.

Diagnostic outcomes varied across testing modalities. Because test selection was guided by phenotype, clinical suspicion, and the availability of diagnostic methods over time, the yields observed for *SCN1A* sequencing, targeted panels, comprehensive panels, and WES should not be interpreted as directly comparable estimates of test performance. Therefore, these yields should not be interpreted as directly comparable performance estimates of the different methods, but rather as real-world diagnostic outcomes within clinically selected testing subgroups.

**Table 1:** Identified pathogenic and likely pathogenic *SCN1A* variants

SCN1A (NM_001165963.4)										
Sex	Age	Age of seizure onset	Age at diagnosis	Sequence change	Amino acid change	Classification# (evidence)	Testing method	Novelty	Segregation status	Clinical phenotype
F	10 years	9 months	5 years	c.272T > C	p.Ile91Thr	Pathogenic (PS2_Strong, PM2_Supporting, PP3_Strong)	Sanger sequencing	Recurrent	de novo	Febrile Seizures
M	17 years	6 months	12 years	c.358del	p.Ile120*	Pathogenic (PS2_Strong, PM2_Supporting, PVS1_Very Strong)	Sanger sequencing	Novel	de novo	Dravet sy
F	4 years	4 months	1 year	c.426T > G	p.Cys142Trp	Likely pathogenic (PS2_Strong, PM2_Supporting, PP3_Moderate)	Sanger sequencing	Novel	de novo	Dravet sy
F	10 years	8 months	3 years	c.548_554del	p.Phe183Serfs*31	Likely pathogenic (PM2_Supporting, PVS1_Very Strong)	Sanger sequencing	Novel	No segregation testing done	Dravet sy
M	7 years	6 months	2 years	c.550 A > C	p.Thr184Pro	Likely pathogenic (PS2_Strong, PM2_Supporting, PP3_Strong)	Sanger sequencing	Novel	de novo	Genetic Epilepsy with Febrile Seizures Plus
M	9 years	1 year	3 years	c.580G > A	p.Asp194Asn	Likely pathogenic (PS2_Strong, PM2_Supporting, PM5_Moderate, PP3_Moderate)	Sanger sequencing	Recurrent	de novo	Genetic Epilepsy with Febrile Seizures Plus
M	6 years	4 months	2 years	c.1130G > A	p.Arg377Gln	Pathogenic (PM2_Supporting, PP3_Strong, PM5_Supporting, PS4_Strong)	Sanger sequencing	Recurrent	No segregation testing done	Dravet sy
M	12 years	3 years	7 years	c.1256T > G	p.Leu419Arg	Likely pathogenic (PM2_Supporting, PP3_Strong, PM1_Moderate)	Sanger sequencing	Novel	No segregation testing done	Genetic Focal Epilepsy – Unclassified
M	19 years	4 months	14 years	c.1261G > C	p.Val421Leu	Likely pathogenic (PM2_Supporting, PP3_Strong, PM1_Moderate, PM5_Moderate)	Sanger sequencing	Novel	No segregation testing done	Genetic Epilepsy with Febrile Seizures Plus
M	5 years	8 months	3 years	c.1834 C > T	p.Arg612*	Pathogenic (PVS1_Very Strong, PM2_Supporting, PS4_Strong, PS2_Strong)	Sanger sequencing	Recurrent	de novo	Dravet sy
M	6 years	1 year	2 years	c.2303 C > T	p.Pro768Leu	Likely pathogenic (PM5_Moderate, PM2_Supporting, PS4_Moderate, PP3_Moderate)	Sanger sequencing	Recurrent	No segregation testing done	Genetic Epilepsy with Febrile Seizures Plus
F	13 years	6 months	5 years	c.2589 + 3 A > T	–	Pathogenic (PVS1_ Strong, PM2_Supporting, PS4_Strong)	Sanger sequencing	Recurrent	No segregation testing done	Dravet sy
F	7 years	6 months	2 years	c.2593 C > T	p.Arg865*	Pathogenic (PVS1_Very Strong, PS2_Strong, PS4_Strong, PM2_Supporting)	Sanger sequencing	Recurrent	de novo	Dravet sy
F	5 years	8 months	1 year	c.2606T > A	p.Leu869*	Pathogenic (PVS1_Very Strong, PM2_Supporting, PS4_Strong)	Sanger sequencing	Novel	No segregation testing done	Dravet sy
F	35 years	9 months	29 years	c.2789 C > T	p.Pro930Leu	Likely pathogenic (PS4_Strong, PM2_Supporting, PP3_Moderate)	Sanger sequencing	Novel	No segregation testing done	Genetic Epilepsy with Febrile Seizures Plus
F	6 years	5 months	5 years	c.2836 C > T	p.Arg946Cys	Pathogenic (PM5_Moderate, PM2_Supporting, PS2_Strong, PS3_Supporting PP3_Strong)	Sanger sequencing	Recurrent	*de novo*	Febrile seizures
M	8 years	6 months	2 years	c.2861 A > G	p.Glu954Gly	Likely pathogenic (PM5_Moderate, PM2_Supporting, PP3_Strong)	Sanger sequencing	Recurrent	No segregation testing done	Genetic Epilepsy – Unclassified
M	8 years	16 months	4 years	c.2928G > A	p.Met976Ile	Likely pathogenic (PS1_Strong, PM2_Supporting, PP3_Moderate)	Sanger sequencing	Recurrent	No segregation testing done	Febrile seizures
M	10 years	7 months	4 years	c.2950 C > G	p.Leu984Val	Pathogenic (PS2_Strong, PM5_Moderate, PM1_Moderate PM2_Supporting, PS4_Moderate, PP3_Moderate)	Sanger sequencing	Recurrent	de novo	Genetic Epilepsy – Unclassified
M	10 years	5 months	3 years	c.2975T > G	p.Leu992Arg	Likely pathogenic (PM1_Moderate PM2_Supporting PM5_Moderate, PP3_Strong)	Sanger sequencing	Novel	No segregation testing done	Febrile seizures
M	20 years	1 year	13 years	c.3343_3344del	p.Val1115Thrfs*8	Pathogenic (PVS1_VeryStrong, PS4_Strong, PM2_Supporting)	Sanger sequencing	Recurrent	No segregation testing done	Dravet sy
F	7 years	6 months	2 years	c.3550 + 2 T > C	–	Pathogenic (PVS1_VeryStrong, PS4_Strong, PM2_Supporting)	Sanger sequencing	Recurrent	No segregation testing done	Genetic Epilepsy – Unclassified
F	11 years	2 years	5 years	c.3610T > C	p.Trp1204Arg	Pathogenic (PS4_Strong, PM2_Supporting, PM5_Moderate, PP3_Strong)	Sanger sequencing	Recurrent	No segregation testing done	Dravet sy
F	3 years	6 months	1 year	c.3661del	p.Glu1221Argfs*7	Likely pathogenic (PVS1_VeryStrong, PM2_Supporting)	Sanger sequencing	Novel	No segregation testing done	Febrile Seizures
F	4 years	1 year	3 years	c.3925 C > T	p.Leu1309Phe	Pathogenic (PS2_Strong, PS4_Strong, PM2_Supporting, PM5_Moderate PP3_Moderate)	Sanger sequencing	Recurrent	de novo	Dravet sy
M	12 years	9 months	7 years	c.3999G > A	p.Met1333Ile	Pathogenic (PS4_Strong, PM1_Moderate, PM2_Supporting, PP3_Strong)	Sanger sequencing	Recurrent	No segregation testing done	Genetic Epilepsy with Febrile Seizures Plus
F	10 years	3 months	2 years	c.4379_4380insAT	p.Phe1461Serfs*5	Pathogenic (PVS1_VeryStrong, PS2_Strong, PM2_Supporting)	Sanger sequencing	Novel	No segregation testing done	Dravet sy
M	11 years	11 months	5 years	c.4547 C > A	p.Ser1516*	Pathogenic (PVS1_VeryStrong, PS2_Strong, PM2_Supporting)	Sanger sequencing	Recurrent	No segregation testing done	Genetic Epilepsy – Unclassified
F	5 years	4 months	2 years	c.4581 + 1G > A	–	Pathogenic (PVS1_VeryStrong, PS2_Strong, PS4_Strong, PM2_Supporting)	Sanger sequencing	Recurrent	de novo	Febrile Seizures
M	7 years	8 months	5 years	c.4582-1G > A	–	Pathogenic (PVS1_VeryStrong, PP3_Moderate, PM2_Supporting)	Sanger sequencing	Recurrent	No segregation testing done	Febrile Seizures
F	13 years	6 months	12 years	c.4602del	p.Phe1535Leufs*4	Likely pathogenic (PVS1_VeryStrong, PM2_Supporting)	Sanger sequencing	Novel	No segregation testing done	Dravet sy
M	15 years	1 year	12 years	c.4888G > A	p.Val1630Met	Likely pathogenic (PS2_Strong, PM2_Supporting, PM5_Moderate, PP3_Moderate)	Sanger sequencing	Recurrent	de novo	Dravet sy
F	4 years	4 months	1 year	c.5012T > G	p.Phe1671Cys	Likely pathogenic (PM1_Moderate PM2_Supporting PP3_Strong)	Sanger sequencing	Novel	No segregation testing done	Genetic Epilepsy with Febrile Seizures Plus
F	18 years	5 months	12 years	c.5038G > A	p.Val1680Ile	Likely pathogenic (PS2_Strong, PM2_Supporting, PM5_Moderate, PP3_Moderate)	Sanger sequencing	Novel	*de novo*	Genetic Epilepsy with Febrile Seizures Plus
F	5 years	7 months	3 years	c.5054 C > A	p.Ala1685Asp	Likely pathogenic (PM2_Supporting, PM5_Moderate, PP3_Strong)	Sanger sequencing	Recurrent	No segregation testing done	Dravet sy
M	22 years	6 months	16 years	c.5339T > C	p.Met1780Thr	Pathogenic (PS2_Strong, PM1_Moderate PM2_Supporting PM5_Moderate, PP3_Strong)	Sanger sequencing	Recurrent	de novo	Febrile seizures

*P* Pathogenic, *LP* Likely pathogenic, *M* male, *F* female; **#**ClinGen Epilepsy Sodium Channel Expert Panel (VCEP) Specifications to the ACMG/AMP Guidelines for *SCN1A* (Version 2.0.0)

**Table 2:** Detected *SCN1A* (NM_001165963.4) variants of uncertain significance

Location	Variant	Classification# (evidence)	Testing method	Novelty	Segregation status	Clinical phenotype
Exon 7	c.542 A > G p.Glu181Gly	VUS (PM2_Supporting, PP3_Supporting)	Seanger sequencing	Recurrent	No segregation testing done	Dravet sy
Exon 14	c.1663T > A p.Ser555Thr	VUS (PM2_Supporting, PP3_Supporting)	Seanger sequencing	Novel	No segregation testing done	Genetic Epilepsy with Febrile Seizures Plus
Exon 14	c.1882T > A p.Ser628Thr	VUS (PM2_Supporting, PS2_Strong)	Seanger sequencing	Recurrent	de novo	Dravet sy
Exon 12	c.1343T > C p.Ile448Thr	VUS (PM2_Supporting, PS2_Strong, BP4_Supporting)	Seanger sequencing	Recurrent	de novo	Developmental and Epileptic Encephalopathy (DEE) – Unclassified
Exon 17	c.2508 C > A p.Asp836Glu	VUS (PM2_Supporting, PP3_Moderate)	Seanger sequencing	Recurrent	No segregation testing done	Dravet sy
Exon 18	c.2747 A > G p.Tyr916Cys	VUS (PP3_Moderate, PM2_Supporting)	Seanger sequencing	Recurrent	No segregation testing done	Febrile Seizures
Exon 18	c.2782 C > A p.Gln928Lys	VUS (PM2_Supporting, PS2_Strong)	Seanger sequencing	Novel	de novo	Genetic Epilepsy – Unclassified
Exon 19	c.3151 C > T p.Leu1051Phe	VUS (PM2*_*Supporting)	Seanger sequencing	Novel	No segregation testing done	Febrile Seizures Plus
Exon 20	c.3440 A > T p.Glu1147Val	VUS (PM2_Supporting, PS2_Strong)	Seanger sequencing	Novel	de novo	Genetic Epilepsy with Febrile Seizures Plus
Exon 21	c.3556G > A p.Val1186Ile	VUS (PM2_Supporting, BP4_Supporting, PS2_Strong)	Seanger sequencing	Recurrent	de novo	Febrile Seizures
Exon 29	c.5326G > A p.Val1776Ile	VUS (PM1_Moderate, PM2*_*Supporting)	Seanger sequencing	Recurrent	No segregation testing done	Genetic Epilepsy – Unclassified
Exon 29	c.5735G > A p.Arg1912Gln	VUS (PM2_Supporting, PM5_Moderate, PP3_Moderate)	Seanger sequencing	Recurrent	No segregation testing done	Dravet sy
Exon 29	c.5785 C > T p.His1929Tyr	VUS (PM2_Supporting, PS2_Strong)	Seanger sequencing	Novel	de novo	Dravet sy

#ClinGen Epilepsy Sodium Channel Expert Panel (VCEP) Specifications to the ACMG/AMP Guidelines for *SCN1A* (Version 2.0.0); *VUS* variants of uncertain significance

The top panel shows an approximate schematic of the NaV1.1 alpha subunit, encoded by the *SCN1A* gene including its four homologous domains (DI–DIV), interdomain linkers, and terminal regions. The bottom panel illustrates the position of pathogenic/likely pathogenic (P/LP) variants and variants of uncertain significance (VUS) across the corresponding protein structure. Novel variants are indicated in bold.

Stacked lollipop plot showing the number and type of germline *SCN1A* variants detected in each exon. Variants are categorized as pathogenic/likely pathogenic (P/LP) and variants of uncertain significance (VUS), enabling visual comparison of their distribution across coding regions. Intronic variants are included and assigned to the exon of which splicing they are predicted to affect.

Regarding next generation sequencing, the most frequently implicated epilepsy-associated genes were *SCN8A*,* PCDH19*,* SLC2A1*,* SLC6A1*, and *CACNA1A* in this study. In total, 15 variants in 12 genes were detected by NGS panel examination. Among these gene variants, nine were novel. Tables 3 and 4 summarize the detected epilepsy gene variants with associated phenotypes identified by NGS-based panel sequencing.

Our findings indicate that the diagnostic yield increases in parallel with the number of genes analysed — from single *SCN1A* test to the application of an extensive epilepsy gene panel. While the yield was 9% when a single gene was analysed, it increased to 14% with a 7-gene epilepsy panel, and the use of a comprehensive 475-gene epilepsy panel resulted in a diagnostic yield of 21%.

WES analysis identified 21 variants in 19 genes. Six of these variants were novel. After parental testing, variants of uncertain significance (in *CHD2*,* SCN8A*, and *SYN1* genes) were proved to be de novo, thus the classification was modified to likely pathogenic. Table 5 presents the summary of clinical characteristics of epilepsy syndrome patients with affected genes.

**Table 3:** Pathogenic and likely pathogenic epilepsy gene variants identified using NGS-based panel testing

Sex	Age	Age at seizure onset	Seizure type/Epilepsy syndrome	EEG abnormalities	Brain MRI finding	Gene	Variant	ACMG classification (evidence)	#OMIM Phenotype (OMIM Inheritance)	Inheritance	Testing method	Novelty
F	11 years	11 months	GTCS	generalized IEDs	negative	*SCN8A* (NM_001330260.2)	c.4912 C > T p.Arg1638Cys	Pathogenic (PS4, PM2, PM1, PP3, PP2, PP5)	Developmental and epileptic encephalopathy 13 #614,558 (AD)	AD	targeted custom NGS panel	Recurrent
F	14 years	18 months	epileptic spasms, tonic	bilateral synchronous and asynchronous occipital IEDs	enlarged ventricles, corpus callosum hypoplasia	*PCDH19* (NM_001184880.2)	c.1682 C > T p.Pro561Leu	Pathogenic (PS4, PM2, PM5, PM1, PP3, PP2, PP5)	Developmental and epileptic encephalopathy 9 #300,088 (XL)	XL	targeted custom NGS panel	Recurrent
F	15 years	20 months	GTCS, tonic	interictal and ictal frontopolar EDs	partial hippocampal sclerosis	*PCDH19* (NM_001184880.2)	c.1152_1180del p.Gln385Serfs*6	Pathogenic (PVS1, PS2, PM2)	Developmental and epileptic encephalopathy 9 #300,088 (XL)	XL	targeted custom NGS panel	Novel
F	7 years	16 months	myoclonic, atonic	slow background activity, generalized spike-and-wave bursts during sleep, pseudo focal IEDs	negative	*SLC2A1* (NM_006516.4)	c.112 A > T p.Lys38*	Pathogenic (PVS1, PS2, PM2)	GLUT1 deficiency syndrome 1, 2 #606,777 #612,126 (AD, AR)	AD	targeted custom NGS panel	Novel
F	21 years	3 years	paroxysmal confusion and eye movements	NR	NR	*SLC2A1* (NM_006516.4)	c.467T > A p.Leu156Gln	Pathogenic (PS2, PP3, PM2, PM1, PP2)	GLUT1 deficiency syndrome 1, 2 #606,777 #612,126 (AD, AR)	AD	targeted custom NGS panel	Novel
M	23 years	6 years	GTCS, eyelid myoclonia	photo stimulation-induced multi-spike wave discharges	brainstem lesion	*CHD2* (NM_001271.4)	c.1886T > A p.Leu629*	Likely pathogenic (PVS1, PM2)	Developmental and epileptic encephalopathy 94 #615,369 (AD)	AD	targeted custom NGS panel	Novel
F	32 years	13 years	GTCS	IEDs from the bitemporal region	negative	*UBTF* (NM_014233.4)	c.1400 C > A p.Ser467*	Pathogenic (PVS1, PM2, PS2)	Neurodegeneration, childhood-onset, with brain atrophy #617,672 (AD)	AD	comprehensive NGS panel	Novel
F	28 years	3 years	GTCS, generalized myoclonic	generalized spike-and wave paroxysms during sleep and arousal	NR	*SETD1B* (NM_001353345.2)	c.3932_3941del p.Pro1311Leufs*2	Likely pathogenic (PVS1, PM2)	Intellectual developmental disorder with seizures and language delay #619,000 (AD)	AD	comprehensive NGS panel	Novel
M	16 years	2 years	myoclonic - atonic	bilateral synchronous spikes	Multiple focal white matter lesions, left hippocampal malrotation	*SLC6A1* (NM_003042.4)	c.860 A > T p.Asp287Val	Pathogenic (PS2, PP3, PM2, PM1, PP2)	Myoclonic-atonic epilepsy #616,421 (AD)	AD	comprehensive NGS panel	Novel
M	14 years	3 years	myoclonic-atonic, tonic	negative	Subcortical cerebral atrophy	*SLC6A1* (NM_003042.4)	c.1366 A > G p.Ser456Gly	Pathogenic (PS2, PM2, PM5, PM1, PP3, PP2)	Myoclonic-atonic epilepsy #616,421 (AD)	AD	comprehensive NGS panel	Novel
M	46 years	2 years	idiopathic generalized	temporo-parieto –occipital region IED with secondary generalised	negative	*CACNA1A* (NM_001127222.2)	c.409G > A p.Glu137Lys	Pathogenic (PP3, PS4, PM2, PP2, PP5)	Developmental and epileptic encephalopathy 42 #617,106 (AD)	AD	comprehensive NGS panel	Recurrent
F	17 years	in utero	EIEE	negative	cerebellar atrophy	*SLC13A5* (NM_177550.5)	c.425 C > T p.Thr142Met	Pathogenic (PM3, PM2, PS3, PP1, PP5)	Developmental and epileptic encephalopathy 25, with amelogenesis imperfect #615,905 (AR)	AR	comprehensive NGS panel	Recurrent
M	15 years	9 months	myoclonic absence	bilateral epileptiform discharges	right frontal and left parietal arachnoid cyst, left peritrigonal white matter lesion	*KCNC1* (NM_001112741.2)	c.1196 C > T p.Thr399Met	Pathogenic (PP3, PS2, PM2, PP2, PS3, PP5)	Epilepsy, progressive myoclonic 7 #616,187 (AD)	AD	comprehensive NGS panel	Recurrent
M	22 years	5 years	GTCS	bilateral IEDs	structural abnormalities of amygdala and hippocampus, pineal germinoma	*UBE2A* (NM_003336.4)	c.3G > A p.Met1Ile	Likely pathogenic (PS1, PM2, PVS1)	Intellectual developmental disorder, X-linked syndromic, Nascimento type #300,860 (XLR)	XLR	comprehensive NGS panel	Novel
M	43 years	3.5 years	tonic, GTCS, Lennox-Gastaut syndrome	repetitive bilateral spike discharges	negative	*TREX1* (NM_033629.6)	c.868_885del p.Pro290_Ala295del	Pathogenic (PM3, PM2, PM4, PP1, PP5)	Aicardi-Goutieres syndrome 1 #225,750 (AD, AR)	AD	comprehensive NGS panel	Recurrent

*NR* not reported, *IED* Interictal Epileptiform Discharges, *ED* Epileptiform Discharges, *EEG* electroencephalogram, *GTCS* Generalized Tonic-Clonic Seizure, *ESES* Electrical Status Epilepticus in Sleep, *EIEE* Early Infantile Epileptic Encephalopathy, *AD* autosomal dominant, *AR* autosomal recessive, *XLR* X-linked recessive, *XL* X-linked, *ACMG* American College of Medical Genetics and Genomics

**Table 4:** Variants of uncertain significance (VUS) detected by NGS-based epilepsy panel testing

Gene	Variant	Amino acid change	ACMG classification (evidence)	OMIM phenotype
*GRIN2A* (NM_001134407.3)	c.2229 A > C	p.Glu743Asp	VUS* (PM2, PM1)	Epilepsy, focal, with speech disorder and with or without impaired intellectual development (#245570)
*HECW2* (NM_020760.4)	c.401 C > T	p.Pro134Leu	VUS (PM2, PP3, PP2)	Neurodevelopmental disorder with hypotona, seizures, and absent language (#617268)
*CUX2* (NM_015267.4)	c.2764_2766del	p.Lys922del	VUS (PM2, PM4)	Developmental and epileptic encephalopathy 67 (#618141)
*MEF2C* (NM_001131005.2)	c.259G > A	p.Ala87Thr	VUS (PM2, PP2)	Neurodevelopmental disorder with hypotonia, stereotypic hand movements, and impaired language (#613443)
*SLC6A5* (NM_004211.5)	c.1795 C > T	p.Arg599Cys	VUS (PM2, PP3)	Hyperekplexia 3 (#614618)
*SETD1B* (NM_001353345.2)	c.1489G > A	p.Asp497Asn	VUS (PM2, PP2)	Intellectual developmental disorder with seizures and language delay (#619000)
*SCN8A* (NM_001330260.2)	c.3137 C > A	p.Ala1046Asp	VUS* (PM2, PP3, PP2)	Developmental and epileptic encephalopathy 13 (#614558)
*SCN9A* (NM_001365536.1)	c.5197 C > T	p.Pro1733Ser	VUS (PM2, PP3)	Generalized epilepsy with febrile seizures plus, type 7 (GEFS + 7) (#613863)
*GABBR2* (NM_005458.8)	c.1925G > T	p.Arg642Leu	VUS (PM2, PP2, BP6)	Neurodevelopmental disorder with poor language and loss of hand skills (#617903)
*CLCN2* (NM_004366.6)	c.508 A > G	p.Ile170Val	VUS* (PP3, PM2)	Epilepsy, idiopathic generalized (#607628)
*PCDH19* (NM_001184880.2)	c.1711G > T	p.Gly571Cys	VUS (PM2, PM1, PP2)	Developmental and epileptic encephalopathy 9 (#300088)
*SLC2A1* (NM_006516.4)	c.1288G > T	p.Gly430Cys	VUS* (PM2, PP3, PM1, PP2)	GLUT1 deficiency syndrome 1, infantile onset, severe (#606777)

*Likely Pathogenic: Classification upgraded from VUS based on confirmed *de novo* status (PS2)

## Discussion

In this retrospective single-centre study, we analysed the diagnostic genetic findings of a clinically selected paediatric-onset epilepsy referral cohort examined over a seven-year period. Rather than providing a population-representative overview of paediatric epilepsy genetics in Hungary, our study captures the real-world evolution of genetic testing at our centre, from SCN1A-focused testing toward broader panel- and exome-based diagnostics. The novelty of our findings is the newly identified pathogenic and likely pathogenic variants in *SCN1A* and additional epilepsy-associated genes in Hungarian population samples. Although these variants are novel, they most likely represent “private” variants specific to the studied families rather than a broader population-wide enrichment.

The 9% diagnostic yield for *SCN1A* P/LP variants in our study is notably higher than the 2.4% reported in large-scale, unselected epilepsy cohorts (e.g., *Truty et al.*, 2019). This discrepancy likely stems from our initial diagnostic workflow, which prioritized *SCN1A* sequencing for patients with high clinical suspicion of Dravet syndrome or GEFS+. However, it is important to note that this 9% yield is significantly lower than the 80–90% diagnostic sensitivity traditionally reported for strictly defined, classic Dravet syndrome cohorts, as well as the ~ 50% yield achieved in developmental and epileptic encephalopathies (DEE) via whole exome sequencing (Palmer 2018). This lower global yield in our early cohort is primarily due to phenotypic dilution; the cohort was not restricted to classic Dravet cases but rather included a heterogeneous group of patients with atypical febrile or infantile-onset seizures where clinicians requested *SCN1A* testing largely to rule out these conditions. Additionally, since early testing relied solely on targeted Sanger sequencing, large exonic deletions or duplications (CNVs), which cause approximately 5–10% of Dravet cases, remained undetected.

This targeted approach in the early stages led to a cohort that was phenotypically enriched compared to general epilepsy populations, yet still broader than a strict Dravet cohort. In contrast, the implementation of wider NGS testing in the later stages allowed us to capture a more heterogeneous patient group, more closely reflecting general paediatric epilepsy populations while simultaneously increasing our overall diagnostic yield from 9% to 14% and 21% via multi-gene panels.

The aetiology of epilepsy is often unknown, and in some cases under- or misdiagnosed. The main causes for this are considerable clinical heterogeneity, overlapping phenotypes, limitations of diagnostic tools, inadequate clinical history and examination, application of an inappropriate test (LaFrance et al. 2013; Scheffer et al. 2017; Thijs et al. 2019; Symonds and McTague 2020). With the spread of new-generation methods, it has become increasingly possible to clarify the molecular background of epilepsies of genetic aetiology. Several factors, such as unusual inheritance patterns, epigenetic mechanisms, incomplete penetrance and mosaicism of the central nervous system, can result in diagnostic challenges in the genetic evaluation of patients (Szalai et al. 2020, 2024; Hadzsiev et al. 2023). The careful selection of the most appropriate genetic test is essential for achieving accurate diagnoses, which in turn can inform prognosis and enhance the quality of life for patients - particularly in therapy-resistant epilepsy, where genetic insights may reveal actionable therapeutic targets (Bayat et al. 2022). This strategic approach is highly relevant for historical cohorts like ours; during the pre-NGS era, targeted Sanger sequencing of *SCN1A* was the only available tool nationwide to screen for Dravet syndrome or GEFS+, inherently missing other associated genes. Today, a comprehensive diagnostic workflow dictates that patients who remain unsolved or present only with a VUS after initial single-gene testing or targeted panels should be systematically reflexed to advanced modalities, such as WGS. Utilizing WGS as a subsequent step is crucial to thoroughly interrogate non-coding regions and expand gene coverage, thereby maximizing diagnostic yield in clinical practice.

The genotype-phenotype correlation is also often unclear in epilepsy patients (Chen et al. 2022). Defects in several genes can cause the same electro-clinical syndrome, and vice versa: variants in one gene can cause phenotypic pleiotropy (e.g. *SCN1A*: Dravet syndrome/GEFS+). Furthermore, a complex genetic background involving multiple interacting genes, as well as the influence of modifier genes, likely contributes to the observed phenotypic variability (de Lange et al. 2020; Chi and Kiskinis 2023).

An illustrative example of phenotypic variability in our cohort is the following case. In fact, interestingly, one of our patients is a 3-year-old girl with febrile epilepsy, whose DNA sample contains two likely pathogenic *SCN1A* variants (c.426 T > G; p.Cys142Trp and c.5012T > G; p.Phe1671Cys). Surprisingly, she is characterized by mild, treatable epilepsy and slightly delayed psychomotor development. She presents a notably mild clinical course, remaining seizure-free for 11 months on a dual anti-seizure medication regimen (valproic acid, topiramate). Although the genomic phase could not be determined due to the lack of parental consent for segregation analysis, this case highlights that certain *SCN1A* variant combinations may result in a less severe phenotype than typically expected. This observation underscores the importance of considering interindividual phenotypic variability even in the presence of multiple potentially pathogenic variants.

There are many other reasons for the molecular diagnostic difficulties of epilepsy. Consequently, NGS-based methods, including targeted gene panels and WES, have become primary diagnostic tools in epilepsy (Mefford 2015; Demos et al. 2019). WES provides a hypothesis-free approach by analyzing all protein-coding regions of the genome, thereby improving diagnostic yield and enabling the identification of rare or previously unrecognized variants. NGS-based approaches also allow the detection of complex sequence alterations and determination of allelic origin (Szalai et al. 2024). Based on our findings, WES should be considered a first-line diagnostic tool in suspected monogenic epilepsies.

Beyond its diagnostic value, genetic testing serves as the cornerstone of precision epilepsy therapy. In our study, molecular findings had direct therapeutic consequences: for instance, identifying *SCN1A* variants prevented the use of contraindicated sodium channel blockers, while an *SLC2A1* diagnosis enabled the timely initiation of a ketogenic diet, the gold standard treatment for GLUT1 deficiency syndrome. Additionally, recognizing *PCDH19*-related epilepsy - characterized by X-linked clusters in females - allows for a more tailored approach, potentially involving emerging treatments like ganaxolone. Importantly, these therapeutic implications remain highly relevant in adulthood. In adult patients with a pediatric-onset history, confirming an *SCN1A* mutation is essential to prevent iatrogenic seizure exacerbation by ensuring the lifelong avoidance of sodium channel blockers. Similarly, the benefits of metabolic or targeted neurosteroid therapies are not age-limited, emphasizing that a genetic diagnosis provides a lifelong roadmap for optimized clinical management and a clearer prognostic outlook for families.

In some cases, exome sequencing is not able to provide a definitive result and is not the most appropriate technical solution when the underlying cause of the disease is low-level mosaicism, epigenetic modification, non-coding region variant, copy number variants or structural rearrangements (Rastin et al. 2023).

While in certain diagnosed syndromes - such as various forms of developmental and epileptic encephalopathies - epilepsy represents the primary clinical manifestation, WES has also enabled the identification of rare disorders in which epilepsy occurs only as a secondary feature within a broader phenotypic spectrum. Examples from our cohort include Helsmoortel–van der Aa syndrome and Schinzel–Giedion midface retraction syndrome, as summarized in Table 5. These findings highlight that in complex syndromes, where epilepsy is not a defining characteristic but rather one of several multi-systemic manifestations, WES offers better diagnostic efficacy compared to targeted gene panel testing, facilitating the recognition of ultra-rare genetic conditions.

The main limitation of our study is the lack of functional validation for the novel variants, as their classification relied on ACMG/AMP and ClinGen *SCN1A*-specific guidelines, in silico prediction tools, and segregation analysis. In addition, the genetic testing strategy was not uniform across the cohort, reflecting the evolution of diagnostic approaches over the study period, with patients undergoing single-gene testing, targeted panels, or whole exome sequencing.

Due to the retrospective and pragmatic nature of this real-world cohort, a precise quantitative breakdown of patients per historical testing modality, as well as systematic segregation data for every single variant, could not be uniformly tracked.

Crucially, genetic testing for a significant portion of patients presenting with infantile-onset or fever-triggered seizures was initiated in the pre-NGS era. During this period, targeted Sanger sequencing of *SCN1A* was the sole available modality nationwide, specifically requested by clinicians to confirm or rule out Dravet syndrome or GEFS+ phenotypes. Consequently, other relevant genes associated with the GEFS+ spectrum (such as *SCN1B*) were not assessed in these early cases, meaning the possibility of missed pathogenic variants in alternative genes cannot be excluded. Another limitation is that parental examination and comprehensive genetic testing were not systematically performed in all *SCN1A*-negative cases, particularly in earlier phases of the study or due to limited availability of parental samples. Furthermore, a limitation of our study is that subsequent WGS could not be performed on unsolved cases or patients with a VUS. In Hungary, WGS is currently not supported by routine clinical reimbursement due to financial constraints, restricting our ability to systematically interrogate non-coding regions and broader genomic data in this cohort. Finally, the relatively small and clinically heterogeneous cohort may limit the generalizability of our findings.

While the expansion of genetic testing is associated with an increased diagnostic yield, it is also accompanied by a growing number of variants of uncertain significance, which remain difficult to classify due to insufficient or inconclusive evidence. As evidenced by our results, as summarized in Tables 2 and 4, several VUS were identified during our investigations via various genetic tests. In the absence of population data, genotype–phenotype correlation, functional and segregation studies, as well as sufficiently strong published evidence, the clear classification of variants as either pathogenic or benign is not possible. As a result, clinical decision-making is significantly hindered. For further evaluation of VUS and unresolved cases, not only is the application of the most comprehensive, state-of-the-art molecular testing methods crucial, but also the annual re-evaluation of data and variants is necessary. The interpretation and communication of such findings to patients must be conducted within the framework of comprehensive pre- and post-test genetic counselling and should be exclusively performed by a qualified clinical geneticist with appropriate expertise (Farmer et al. 2021).

In the diagnostic process of epilepsy and other similarly heterogeneous disorders, effective interdisciplinary collaboration is critical. The concerted efforts of the neurologist assessing the patient, the clinical geneticist responsible for pre- and post-test counseling, the molecular biologist conducting and interpreting the genetic analyses, and the bioinformatician processing and analysing sequencing data are essential to ensure accurate and meaningful diagnostic outcomes.

Further genotype-phenotype and functional studies are required to provide more detailed characterization of epilepsy disorder to provide accurate molecular diagnoses for patients with genetically determined epilepsy, thereby paving the way for personalized therapy and genetic counseling, with the primary aim of improving the quality of life of this patient group.

**Table 5:** Genetic aetiologies and corresponding ILAE epilepsy syndromes identified by exome sequencing in our patient cohort

Sex	Age	Age at seizure onset	Clinical features of the patient	Affected gene	Variant(s)	Amino acid change(s)	ACMG Classification	ILAE 2022 Epilepsy Syndrome	OMIM Phenotype (Inheritance)
F	8 year	2 year	Delayed development, seizures, sensorineural hearing loss, hypotonia	*SPATA5* NM_145207.3	c.989_991del	p.Thr330del	Pathogenic (PM3, PM2, PM4, PP5)	Developmental and Epileptic Encephalopathy (DEE) – Unclassified (Childhood-onset)	Neurodevelopmental disorder with hearing loss, seizures, and brain abnormalities #616,577 (AR)
					c.1528 C > T	p.Arg510*	Pathogenic (PVS1, PM3, PM2, PP5)		
F	13 year	5 year	Severe ID with behavioural and language impairment, ASD, dyscrania, dysmorphic features, seizures, MRI abnormalities (dysplasia of corpus callosum, PVL)	***ADNP*** **NM_001282531.3**	**c.1223dup**	p.Ser409Valfs*31	Pathogenic (PVS1, PS2, PM2)	Developmental and Epileptic Encephalopathy (DEE) – Unclassified (Childhood-onset)	Helsmoortel-van der Aa syndrome #615,873 (AD)
F	6 year	18 month	ID, skeletal abnormalities, dysmorphic features, seizures	*SETBP1* NM_015559.3	c.2602G > A	p.Asp868Asn	Pathogenic (PS4, PS2, PM2, PM5, PM1, PP5)	Developmental and Epileptic Encephalopathy (DEE) – Unclassified (Childhood-onset)	Schinzel-Giedion midface retraction syndrome #269,150 (AD)
F	15 year	6 year	Autistic and dysmorphic features, mild ID, seizure	***PCDH19*** **NM_001184880.2**	**c.830_831delinsAA**	p.Phe277*	Pathogenic (PVS1, PM2, PS2)	*PCDH19* clustering epilepsy (Childhood-onset)	Developmental and epileptic encephalopathy 9 #300,088 (XL)
M	50 year	12 month	Seizures. severe ID, feeding difficulties, self-injurious behaviour, psychomotor regression	*CHD2* NM_001271.4	c.1349 C > T	p.Ser450Leu	Likely Pathogenic (PS2, PM2, PP2)	*CHD2*-related epilepsy	Developmental and epileptic encephalopathy 94 #615,369 (AD)
F	11 year	4 year	Progressive neurodegeneration, delay and regression in psychomotor and speech development, seizure	*MFSD8* NM_001371596.2	c.881 C > A*	p.Thr294Lys	Pathogenic (PM3, PM2, PP2, PS3, PP5)	Progressive Neurological Deterioration with Epilepsy	Ceroid lipofuscinosis, neuronal, 7 #610,951 (AR)
M	4 year	in utero	Severe, early epileptic encephalopathy, lack of psychomotor development	***SCN2A*** **NM_001040142.2**	**c.4945 C > G**	**p.Leu1649Val**	Likely Pathogenic (PM1, PM2, PM5, PP3)	*SCN2A*-related developmental and epileptic encephalopathy	Developmental and epileptic encephalopathy 11 #613,721 (AD)
M	15 year	4 year	Hyperekplexia	*SCN8A* NM_014191.4	c.5042G > C	p.Gly1681Ala	Likely Pathogenic (PS2, PM2, PP3)	*SCN8A*-related epilepsy	Myoclonus, familial, 2 #618,364 (AD)
F	14 year	2 year	Epilepsy, autistic features, macrocephaly	*ARHGEF9* NM_001353921.2	c.331 C > T	p.Arg111Trp	Pathogenic (PM3, PP3, PM2, PM5, PP5)	Developmental and Epileptic Encephalopathy (DEE) – Unclassified (Childhood-onset)	Developmental and epileptic encephalopathy 8 #300,607 (XL)
F	4 year	3 month	Global developmental delay, hypotonia, microcephaly, facial dysmorphism corpus callosum agenesis, seizure	*PDHA1* NM_000284.4	c.707 C > A	p.Ala236Glu	Pathogenic (PS2, PS4, PM2, PM1, PP3, PP2, PP5)	Infantile Epileptic Spasms Syndrome (IESS) secondary to metabolic etiology	Pyruvate dehydrogenase E1-alpha deficiency #312,170 (XLD)
F	13 year	3 year	Epileptic encephalopathy, neurodevelopmental disorder, impaired speech, absence seizures	*SLC6A1* NM_003042.4	c.919G > A	p.Gly307Arg	Pathogenic (PS2, PM2, PM1, PP3, PP2, PP5)	Epilepsy with Myoclonic-Atonic Seizures (EMAtS)	Myoclonic-atonic epilepsy #616,421 (AD)
M	13 year	3 year	Developmental regression, stereotypic movements, brain abnormality, ASD, seizure	*STXBP1* NM_001032221.6	c.1095_1096del	p.Cys366Profs*13	Pathogenic (PVS1, PM2, PS4, PP5)	Developmental and Epileptic Encephalopathy (DEE) – Unclassified (Childhood-onset)	Developmental and epileptic encephalopathy 4 #612,164 (AD, AR)
M	44 year	18 month	Delayed psychomotor development, profound intellectual disability, Lennox-Gastaut syndrome	*GABRB2* NM_001371727.1	c.902 A > G	p.Tyr301Cys	Pathogenic (PS2, PM2, PM5, PM1, PP3, PP2, PP5)	Lennox-Gastaut syndrome (LGS)	Developmental and epileptic encephalopathy 92 #617,829 (AD)
F	15 year	2 year	Corpus callosum agenesis, minor anomalies, delayed psychomotor development, absent speech, microcephaly, seizure	***ZEB2*** **NM_014795.4**	**c.2286del**	p.Lys762Asnfs*25	Likely Pathogenic (PVS1, PS2, PM2)	Developmental and Epileptic Encephalopathy (DEE) – Unclassified (Childhood-onset)	Mowat-Wilson syndrome #235,730 (AD)
F	13 year	4 year	Delayed psychomotor development, hypotonia, poor speech, ASD, ADHD, mild ID, seizures	*KCNB1* NM_004975.4	c.934 C > T	p.Arg312Cys	Pathogenic (PS2, PM2, PM5, PM1, PP3, PP2, PP5)	Developmental and Epileptic Encephalopathy (DEE) – Unclassified (Childhood-onset)	Developmental and epileptic encephalopathy 26 #616,056 (AD)
F	20 year	4 year	MRI abnormalities, seizure, learning difficulties	*SYN1* NM_006950.3	c.1799 C > T	p.Pro600Leu	Likely Pathogenic (PS2, PM2)	*SYN1*-related epilepsy	Epilepsy, X-linked 1, with variable learning disabilities and behavior disorders #300,491 (XL)
F	14 year	3 year	Profound delayed psychomotor development, brain abnormalities, seizure	*AARS1* NM_001605.3	c.988 C > T	p.Arg330*	Pathogenic (PVS1, PM2, PS4, PP5)	Developmental and Epileptic Encephalopathy (DEE) – Unclassified (Childhood-onset)	Developmental and epileptic encephalopathy 29 #616,339 (AR)
					c.2738G > A	p.Gly913Asp	Likely Pathogenic (PM2, PS4, PS3, PP1, PP5)		
M	5 year	8 month	Global developmental delay, minor anomalies, brain abnormality, generalized hypotonia, lack of visual and auditory attention, seizure	***DYNC1H1*** **NM_001376.5**	**c.10267G > C**	**p.Ala3423Pro**	Likely Pathogenic (PS2, PM2, PM5, PP3, PP2)	Developmental and Epileptic Encephalopathy (DEE) – Unclassified (Infantile-onset)	Cortical dysplasia, complex, with other brain malformations 13 #614,563 (AD)
F	12 year	3 year	Microcephaly, moderate ID, developmental regression, minor anomalies, intractable epilepsy	***SYNGAP1*** **NM_006772.3**	**c.2973_2979del**	p.Val992Serfs*83	Likely Pathogenic (PVS1, PM2)	Developmental and Epileptic Encephalopathy (DEE) – Unclassified (Childhood-onset)	Intellectual developmental disorder, autosomal dominant 5 #612,621 (AD)

*ASD* Autism Spectrum Disorder, *ADHD* Attention Deficit Hyperactivity Disorder, *ID* Intellectual Disability, *AD* autosomal dominant, *AR* autosomal recessive, *MRI* Magnetic Resonance Imaging, *XL* X-linked, *XLD* X-linked dominant, *GTCS* Generalized Tonic-Clonic Seizure, *: Homozygous; *F* Female, *M* Male

## Supplementary Information

Below is the link to the electronic supplementary material.


Supplementary Material 1


## Data Availability

The datasets generated during the current study are available from the corresponding author on reasonable request.
