# Supplementary material for: From targeted SCN1A analysis to whole exome sequencing: clinical utility and novel genetic findings in a Hungarian paediatric epilepsy cohort
Source: Hum Genet. 2026 Aug 29;145(1):72. doi: 10.1007/s00439-026-02867-w (PMC13526097; doi:10.1007/s00439-026-02867-w)
Supplement: Supplementary file 1 [file 439_2026_2867_MOESM1_ESM.doc]

Appendix 1

Software tools applied in NGS data processing and interpretation

| **Software** | **Version** | **References** |
| --- | --- | --- |
| BWA-MEM | v0.7.17 | ^35^ |
| SAMtools | v1.10 | ^36^ |
| GATK4 | v4.1.8.0 | ^37,38^ |
| ANNOVAR | 2018Apr16 | ^39^ |
| SnpEff | v4.3r | ^40^ |
| SnpSift | v4.3r | ^40^ |
| Franklin (Genoox) | online | [https://franklin.genoox.com](https://franklin.genoox.com/) |
| VarSome | online | ^41^ |

Appendix 2

Gene content of applied targeted custom and comprehensive epilepsy panel.

Next generation sequencing-based targeted custom epilepsy panel*:*

*SCN1A, SCN8A, ARX, CHD2, PCDH19, SLC2A1, STXBP1.*

Comprehensive epilepsy panel:

*AARS1, AARS2, ABAT, ABCA2, ABCD1, ACTL6B, ACY1, ADAM22, ADAR, ADNP, ADPRHL2, ADSL, AFG3L2, AGA, AIFM1, AIMP1, ALDH3A2, ALDH5A1, ALDH7A1, ALG13, ALG6, ALKBH8, AMACR, AMT, ANKRD11, AP2M1, AP3B2, AP4B1, AP4E1, AP4M1, AP4S1, APOPT1, ARG1, ARHGEF9, ARID1B, ARSA, ARV1, ARX, ASAH1, ASNS, ASPA, ASXL3, ATAD1, ATP13A2, ATP1A1, ATP1A2, ATP1A3, ATP6V1A, ATRX, BCKDK, BRAT1, BTD, C12ORF57, CACNA1A, CACNA1B, CACNA1D, CACNA1E, CACNA1G, CACNA1H, CACNA2D2, CACNB4, CAD, CAMK2B, CARS2, CASK, CASR, CC2D1A, CDK9, CDKL5, CERS1, CHD2, CHRNA2, CHRNA4, CHRNB2, CLCN2, CLCN4, CLN3, CLN5, CLN6, CLN8, CLTC, CNKSR2, CNPY3, CNTNAP2, COA7, COL4A1, COL4A2, COL4A3BP, COQ2, COQ4, COX15, COX6B1, CPLX1, CPT2, CSF1R, CSNK2B, CSTB, CTC1, CTSD, CTSF, CUL4B, CUX2, CYFIP2, CYP27A1, D2HGDH, DARS, DARS2, DCX, DDC, DDX3X, DEAF1, DEGS1, DENND5A, DEPDC5, DHDDS, DHFR, DHPS, DIAPH1, DMXL2, DNAJC5, DNM1, DNM1L, DOCK7, DOLK, DPAGT1, DPM1, DPM2, DPYD, DPYS, DYNC1H1, DYRK1A, EARS2, ECHS1, ECM1, EEF1A2, EFHC1, EIF2B1, EIF2B2, EIF2B3, EIF2B4, EIF2B5, EIF3F, EML1, EPM2A, EPRS, ETFA, ETFB, ETFDH, ETHE1, FA2H, FAM126A, FAR1, FARS2, FDFT1, FDX1L, FGF12, FH, FKTN, FLNA, FOLR1, FOXG1, FOXRED1, FRRS1L, FUT8, GABBR2, GABRA1, GABRB1, GABRB2, GABRB3, GABRG2, GALC, GAMT, GCDH, GCH1, GCSH, GFAP, GFM1, GFM2, GJC2, GLB1, GLDC, GLRB, GLS, GLUD1, GNAO1, GNB1, GNE, GOLGA2, GOSR2, GPAA1, GPHN, GRIA3, GRIA4, GRIK2, GRIN1, GRIN2A, GRIN2B, GRIN2D, GRN, GTPBP3, GUF1, HACE1, HCN1, HCN2, HECW2, HEPACAM, HIBCH, HNRNPU, HSD17B10, HSPD1, HTRA1, HTT, IBA57, ICK, IER3IP1, IFIH1, IQSEC2, IRF2BPL, ITPA, KCNA1, KCNA2, KCNB1, KCNC1, KCNH1, KCNJ10, KCNMA1, KCNQ2, KCNQ3, KCNQ5, KCNT1, KCNT2, KCTD3, KCTD7, KDM5C, KIAA1715, KIAA2022, KIF1A, KIF5A, KIF5C, KMT2E, L2HGDH, LGI1, LIAS, LMNB1, LMNB2, LRPPRC, LYRM7, MACF1, MAGI2, MARS2, MBD5, MBOAT7, MDH2, MECP2, MED12, MED17, MEF2C, MFSD8, MIPEP, MLC1, MOCS1, MOCS2, MRPL44, MTFMT, MTHFR, MTOR, NACC1, NBEA, NDST1, NDUFAF3, NDUFAF5, NDUFAF6, NDUFS2, NDUFS4, NDUFS6, NDUFS7, NDUFS8, NDUFV1, NECAP1, NEU1, NEUROD2, NFU1, NHLRC1, NKX6-2, NOTCH3, NPRL2, NPRL3, NR2F1, NRXN1, NSDHL, NT5C2, NTRK2, NUBPL, NUS1, OCLN, OFD1, OPHN1, P4HTM, PACS1, PACS2, PAFAH1B1, PARS2, PCDH19, PEX1, PEX10, PEX12, PEX13, PEX14, PEX16, PEX19, PEX2, PEX26, PEX3, PEX5, PEX6, PGK1, PHACTR1, PHF6, PIGA, PIGB, PIGC, PIGG, PIGN, PIGO, PIGP, PIGQ, PIGS, PIGT, PIGV, PIGW, PITRM1, PLAA, PLCB1, PLP1, PNKP, PNPO, POLG, POLR3A, POLR3B, PPP2CA, PPP3CA, PPT1, PRICKLE1, PRICKLE2, PRIMA1, PRODH, PROSC, PRRT2, PRUNE, PSAP, PSAT1, PTPN23, PTS, PUM1, PURA, PYCR2, QARS, QDPR, RAB11A, RAB11B, RAB39B, RALA, RARS, RELN, RHOBTB2, RMND1, RNASEH2A, RNASEH2B, RNASEH2C, RNASET2, RNF13, RNF216, ROGDI, RORA, RORB, RUSC2, SAMHD1, SCARB2, SCN1A, SCN1B, SCN2A, SCN3A, SCN8A, SCN9A, SCO1, SDHAF1, SERAC1, SERPINI1, SETBP1, SETD1B, SGSH, SIK1, SLC12A5, SLC13A5, SLC19A3, SLC1A2, SLC1A4, SLC25A1, SLC25A15, SLC25A22, SLC25A42, SLC2A1, SLC35A1, SLC35A2, SLC39A8, SLC46A1, SLC6A1, SLC6A5, SLC6A8, SLC9A6, SMARCA2, SMC1A, SMS, SNAP25, SNORD118, SOX10, SPATA5, SPTAN1, SPTBN4, SSR4, ST3GAL3, ST3GAL5, STRADA, STX1B, STXBP1, SUMF1, SUOX, SYN1, SYNGAP1, SYNJ1, SZT2, TAF1, TANGO2, TBC1D20, TBC1D24, TBCD, TBCE, TBCK, TBL1XR1, TCF4, TK2, TPK1, TPP1, TRAK1, TREX1, TRIM8, TRIT1, TSC1, TSC2, TSFM, TTC19, TUBA1A, TUBB2A, TUBB2B, TUBB4A, UBA5, UBE2A, UBE3A, UBTF, UNC80, VAMP2, VARS, VPS13A, WARS2, WASF1, WDR26, WDR45, WWOX, YWHAG, YY1, ZDHHC9, ZEB2, ZFYVE26, ZNHIT3, ZSWIM6.*
